# Medication count, including statin or metformin use, is not associated with influenza vaccine responses in older adults

**DOI:** 10.1016/j.vaccine.2025.127913

**Published:** 2025-10-29

**Authors:** Victoria Li, Heather McClure, Radha Patel, George A. Kuchel, Chris P. Verschoor

**Affiliations:** a University of Connecticut School of Medicine, Farmington, CT, USA; b UConn Center on Aging, University of Connecticut School of Medicine, Farmington, CT, USA; c Health Sciences North Research Institute, Sudbury, ON, Canada; d NOSM University, Sudbury, ON, Canada; e Department of Medicine, McMaster University, ON, Canada

**Keywords:** Vaccination, Medication use, Aging population, Immunogenicity, Standard dose, Polypharmacy

## Abstract

**Background:**

Vaccination helps prevent infections and associated sequelae, especially in older adults. Since the degree to which vaccine responses are influenced by medications is not well described, we examined the association of medication count, including statin or metformin use, with influenza vaccine responsiveness.

**Methods:**

A secondary analysis of data from 542 participants aged 65 years and older randomized to receive either the standard or high dose vaccine during four influenza seasons (2015–2018) was conducted. Medication counts, as well as statin and metformin usage, were self-reported. Associations between vaccine antibody titer responses and medication usage were estimated using mixed model linear regression.

**Results:**

Participants reported taking a median of five medications, with 45 % (*n* = 244) of participants using a statin and 12 % (*n* = 65) metformin. Medication use was higher with frailty, chronic condition count and in males. Our data showed that medication use was not broadly associated with vaccine responsiveness, which held after adjusting for frailty and number of chronic conditions, and when investigated within sex and vaccine dose strata. However, within diabetics, the strong boosting effect of the high-dose vaccine was significantly dampened for metformin users (*n* = 64) as compared to non-users (*n* = 30), but still remained slightly higher than standard-dose responses. This dampening effect was evident in analyses where all antigen-specific responses were combined (i.e. maxRBA, *p* = 0.002), and for responses against seasonal A/H3N2 (*p* = 0.007) and B (*p* = 0.03) antigens.

**Conclusion:**

Vaccine responses varied little with medication usage among older adults. However, our findings suggest metformin may be associated with reductions in the high-dose vaccine response within diabetics.

## 1. Introduction

Influenza is a significant respiratory virus causing between 100,000 and 710,000 hospitalizations and 4900–51,000 deaths per year in the US [1]. Older adults are not only more likely to experience severe outcomes of influenza requiring hospitalization and be at higher risk of secondary infections such as pneumonia, but also comprise approximately 90 % of deaths attributable to influenza [2]. Furthermore, older adults are more likely to suffer both short- and long-term effects of their infection. For example, one study reported a six-fold increase in myocardial infarction risk and a three-fold increase in stroke risk one week after influenza diagnosis [3], while another found that 35 % of older adults hospitalized with influenza were not able to return to their prior level of functioning after discharge, and that the proportion of patients declining after discharge significantly increased with age [2,4].

Annual influenza vaccination is a critical prevention strategy and several have been approved for use in patients 65 years and older. In the US there are three recommended vaccines available for older adults: Flublok (Sanofi Pasteur), a recombinant vaccine with three times the amount of influenza antigen than the standard dose (SD) influenza shot, FLUAD (Seqirus), an adjuvanted SD influenza vaccine, and Fluzone High Dose (Sanofi Pasteur), a high dose (HD) vaccine which contains four times the amount of hemagglutinin antigen as compared to the SD vaccine [5]. While it is known that vaccine efficacy is variable among individuals, the major determinants have yet to be fully elucidated. A 2021 meta-analysis found that in participants who received the influenza vaccine, there was a 26 % reduction in odds of ICU hospitalization and a 31 % reduced risk of death among adults with influenza-associated hospitalization compared to those who were unvaccinated [6]. Factors contributing to vaccine efficacy can include host factors (e.g. age, sex, BMI, and comorbidities), vaccine factors (e.g. type, dose, and product), as well as lifestyle factors (e.g. alcohol consumption, smoking, and exercise) [7]. Factors that are especially variable with age, such as frailty, functional status, and medication use, have yet to be studied in depth.

The role of medication usage in altering vaccine responses is particularly intriguing, given that older adults frequently take at least one medication, with up to 36 % taking five or more [8]. However, previous studies are conflicting with regard to the association of medication usage with vaccine efficacy [9–11]. For example, metformin, an antidiabetic drug that mainly works through inhibiting hepatic gluconeogenesis and increasing insulin sensitivity, is commonly prescribed to older adults and has previously been associated with an increase in influenza vaccine response, decreased T cell exhaustion, and decreased influenza-related hospitalizations and mortality [9,11]. Statins, which lower cholesterol by inhibiting HMG-coA reductase and has been shown to exhibit additional anti-inflammatory properties, represent another common class of medication among older adults that have been shown to potentially be associated with changes in influenza vaccine efficacy [12]. However, observations are variable, ranging from not having a significant association with vaccine efficacy, to decreasing efficacy [12–14]. Given the limited data published on vaccine efficacy while taking certain medications and the lack of studies including both SD and HD influenza vaccines, we sought to examine the association between medication usage, particularly metformin and statins, with influenza vaccine antibody responses in older adults. This secondary analysis of 542 participants included individuals over 65 years old and spanned four consecutive influenza seasons, between 2015 and 2018.

## 2. Methods

### 2.1. Study design

We conducted a secondary analysis of data on adults aged 65 years and older who were vaccinated in the years 2015–2018. From 2015 to 2017, participants took part in a randomized trial to compare the immunogenicity of the SD and HD influenza vaccines (NCT: 01427309), while in 2018 all participants were administered the HD vaccine. The study design and findings from the randomized study have been previously published [15,16]. Participants were recruited through the UConn Center on Aging Recruitment Core from communities belonging to and surrounding Hartford, Connecticut (UCHC), and through the Health Sciences North Research Institute (HSNRI) from the community of Greater Sudbury, Ontario, Canada. Inclusion criteria included the following: at least 65 years old and vaccinated in the previous influenza season. Exclusion criteria included the following: known immunosuppressive disorders or medications including prednisone in doses > 10 mg/day, previous severe reaction to the vaccine, egg, latex, or thimerosal allergies, or refusal of vaccination. A randomized, double-blind design was used to assign study participants to receive a SD or HD (Fluzone) influenza vaccine. Research coordinators ensured that vaccinations were scheduled at least two weeks after any acute respiratory illness. Over the four years, a total of 542 participants were studied, and participants were allowed to take part in additional years if interested. The parent study and this secondary analysis was approved by the University of Connecticut Health Institutional Review Board (#15–006–3) and Health Sciences North Research Ethics Board (#985).

### 2.2. Participant characteristics

Age, sex, body mass index (BMI), frailty, chronic conditions, cytomegalovirus (CMV) serostatus, and medication history were obtained from study participants at their initial visit in each year of the study. BMI was categorized according to World Health Organization guidelines into normal/underweight (≤24.9), overweight (25–29.9), and obese (≥30). Frailty was measured using the Fried frailty phenotype which evaluated five characteristics: shrinking (weight loss), weakness (grip strength), endurance and energy (exhaustion), slowness (time to walk), and physical activity level (kilocalories expended). The presence of three or more of these characteristics indicated frailty [17]. Fried frailty was then further categorized into robust (score of 0), pre-frail (1–2), and frail (3 or more). The following chronic conditions were recorded and summed as a total chronic condition count: diabetes, angina, arrhythmia, valvular disorder, previous myocardial infarction, heart failure, peripheral vascular disease, hyperlipidemia, hypertension, cerebrovascular, asthma, chronic obstructive pulmonary disease, renal failure, nephrotic syndrome, dementia, cerebral palsy, hemiplegia, seizures, Parkinsons disease, hepatic cirrhosis, inflammatory bowel disease, peptic ulcers, gastroesophageal reflux disease, prior cancer diagnosis, lupus, connective tissue disorder, depression, and anxiety, bipolar, or psychotic disorders. CMV serostatus was determined in serum prior to vaccination using a CMV IgG ELISA kit (Genesis Diagnostics Inc., Cambridgeshire, UK) according to the manufacturer’s instructions.

### 2.3. Medication usage

Information regarding prescribed and over-the-counter medications and health supplements was collected during participant interviews. Participants were asked to bring their medications, and a research coordinator recorded the name, dose, and interval of each. For this study we only considered medications either prescribed by a physician or commonly directed for use by a physician to treat an illness. This did not include supplements (e.g. vitamins and probiotics) or nutraceuticals (e.g. glucosamine and fish oil). The total medication count was categorized as 0–4, 5–9 and 10 or more, as per previous literature [18], and reported metformin or statin usage was categorized as yes/no. Medications were classified according to the FDA General Drug Categories [19].

### 2.4. Vaccine antibody responses

Hemagglutination inhibition antibody titers were used to measure immunogenicity against three of the strains shared across both SD and HD vaccines: influenza A/H1N1 and A/H3N2 and Influenza B/Victoria. For each year, this included the following antigens: 2015 – A/California/7/2009 (H1N1), A/Switzerland/9715293/2013 (H3N2), and B/Brisbane/60/2008; 2016 – A/California/7/2009 (H1N1), A/Hong Kong/4801/2014 (H3N2), and B/Brisbane/60/2008; 2017 – A/Michigan/45/2015 (H1N1), A/Hong Kong/4801/2014 (H3N2), and B/Brisbane/60/2008; 2018 – A/Michigan/45/2015 (H1N1), A/Singapore/INFIMH-16–0019/2016 (H3N2), and B/Colorado/06/2017. Blood was drawn at time of vaccination, then at four weeks post-vaccination. Laboratory testing was conducted after each study year, and participant serum was randomized before plating. Vaccine efficacy was categorized two different ways based on analyses from a previous study [16]. Data was quantified as a metric of Maximum Residual after Baseline Adjustment (maxRBA), which represents the change in antibody response across all strains tested for a given year in standardized units [20]. For example, a participant with a maxRBA of 1 exhibited an average antibody response that was 1 standard deviation above the cohort mean for that year; a participant with a maxRBA of − 1 exhibited and antibody response that was 1 standard deviation below the cohort mean. Antigen-specific antibody responses were expressed as the log 4-week post-vaccination titer relative to pre-vaccination.

### 2.5. Statistical analysis

Participant characteristics were summarized as mean (standard deviation) and median (range) for continuous variables and count (frequency) for categorical variables. Bivariate analyses to compare total medication count across different age, sex, or frailty groups were performed by Wilcoxon rank-sum test, whereas comparison of metformin or statin use was performed by chi-square or Fisher’s exact test (where applicable). For chi-square or Fisher’s exact test results of *p* < 0.10, post-hoc comparisons were performed using multinomial regression. Associations between medication usage and vaccine antibody response (i.e. maxRBA or 4-week response relative to baseline) were estimated by mixed model linear regression. Two models were employed: the first including only the fixed effect of medication and random intercepts for site, year and participant, while the second also included the fixed effects of age, sex, vaccine dose, frailty, chronic condition count and CMV serostatus; models including the standardized log 4-week antibody titer as the outcome also include the log baseline antibody titer as a fixed effect, regardless of other covariates. Interaction analysis was performed to investigate the modifying effect of sex and vaccine dose on associations between medication usage and vaccine responses. Here, linear mixed models were repeated with a sex or dose by medication multiplicative interactive included as a fixed effect; results are presented as the estimated marginal means and 95 % confidence interval of vaccine response within medication categories for females and males or SD and HD recipients.

All analyses were performed in R version 4.3.2.

## 3. Results

### 3.1. Patient characteristics and medication usage

In this study, 542 participants were analyzed across four years (Table 1). The average age was 77 years, with 67 % of the study population being female. The predominant BMI was overweight, representing 41 % of the study participants, while 30 % were normal/underweight and 28 % obese. Approximately 60 % of the participants were considered pre-frail according to the Fried frailty phenotype, followed by robust and frail for 32 % and 9 % respectively. Approximately 85 % of participants reported at least 1 chronic condition, with 17 %, 46 %, and 56 % reporting diabetes, hyperlipidemia or hypertension. Roughly half were seronegative for CMV and SD influenza vaccine was given to 48 % of participants while 52 % received the HD vaccine. Of note, only the HD vaccine was given during the study in 2018, and all participants were recruited from UCHC.

**Fig. 1.**
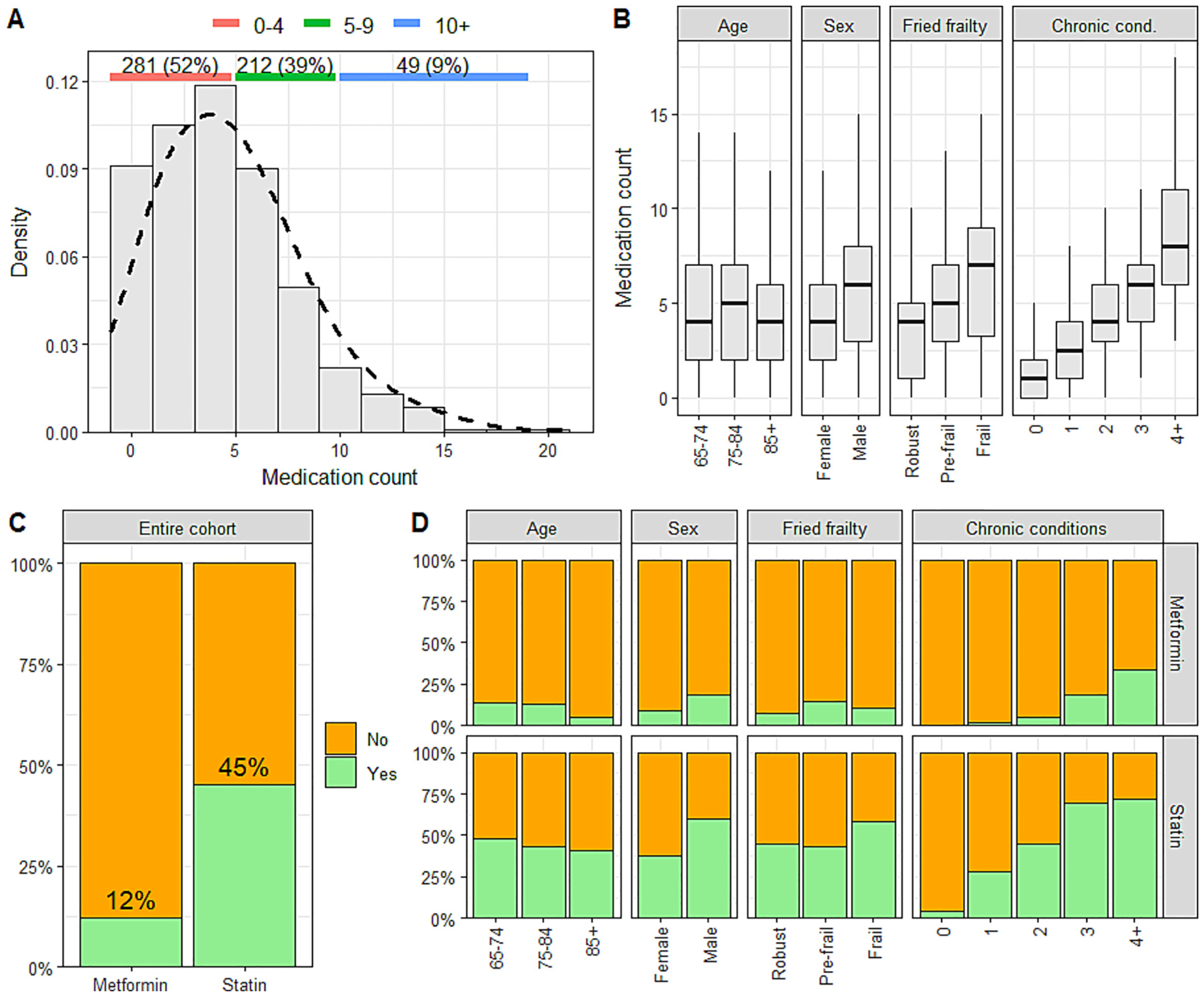
Descriptive analysis of study characteristics. (A) Histogram of total medications reported for each participant. (B) Box and whisker plots of medication count stratified by age, sex, and Fried frailty phenotype. (C) Percentage of participants taking metformin and statins. (D) Percentage taking metformin and statins stratified by age, sex, and Fried frailty phenotype. Asterisks denote a significant difference relative to the reference category (i.e. 65–74, female or robust): ***, *p* < 0.001; **, *p* < 0.01; *, *p* < 0.05.

Over the entire study, 2602 medications were reported, representing 34 drug classes (Supplementary Table 1). The most frequent classes were antihypertensives (*n* = 349, 13.4 %), antithrombic agents (*n* = 331, 12.7 %), and lipid lowering agents (*n* = 295, 11.3 %); roughly half of all participants reported taking at least one of these medications. Overall, participants reported taking a median of 5 medications (minimum = 0, maximum = 18), and 52 %, 39 %, and 9 % of participants reported taking 0–4, 5–9, 10+ medications, respectively (Fig. 1). Only 32 (5.9 %) did not report taking any medications. Compared to participants who were aged 65–74, total medication usage was higher in participants aged 75–84 [4.8 vs 5.0, *p* = 0.8] but lower in participants aged 85+ [4.8 vs 4.4, p = 0.8], although this failed to reach significance. However, medication count was significantly higher in males than females [5.7 vs 4.4, *p* < 0.001]. As expected, medication usage was significantly higher for frail as compared to pre-frail [6.8 vs 5.1, p < 0.001] and robust participants [6.8 vs 3.8, p < 0.001] and increased significantly with number of chronic conditions (p < 0.001 for all comparisons).

Of the specific medications taken, 12 % of study participants reported taking metformin and 45 % a statin. Metformin usage was higher in ages 65–74 (10 %) and 75–84 (10 %) compared to 85+ (5 %) year olds, although not significantly different. Similarly, statin usage was higher in ages 65–74 (48 %), followed by 75–84 (43 %) and 85+ (41 %). More males reported taking a metformin (14 vs. 7 %, *p* < 0.001) or statin (60 vs. 37 %, p < 0.001) as compared to females. About 11 % of participants in the frail and pre-frail groups were taking metformin while only 6 % of the robust group was taking this medication. As for statin use, a larger proportion of frail participants were taking a statin (59 %), followed by robust (45 %), and then prefrail (43 %). Significant differences were not apparent for either drug. Metformin and statin use were significantly higher with increased chronic conditions, and both were much more prevalent for their related indications. Only 1 of 448 participants with diabetes reported taking metformin, in contrast to 64 of 94 (68 %) of reported diabetics (*p* < 0.001). For those reporting hyperlipemia, 221 of 251 (88 %) also reported taking statins, as compared to only 8 % without disease (p < 0.001). For those reporting hypertension, 155 of 303 (51 %) also reported taking statins, as compared to 37 % without disease (*p* = 0.002).

### 3.2. Associations between medication usage and vaccine responsiveness

**Fig. 2.**
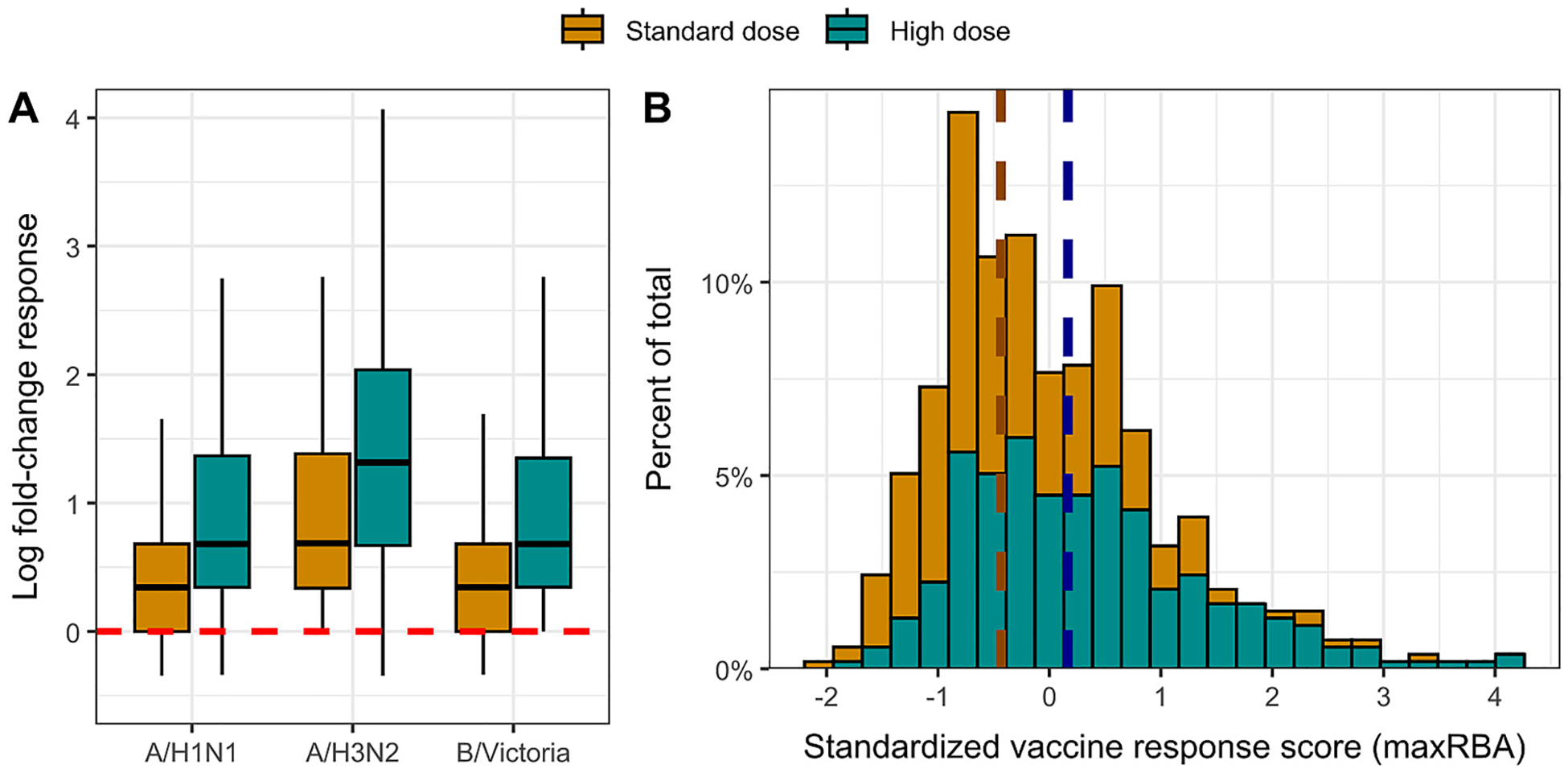
Influenza vaccine responses stratified by dose. (A) Log fold-change vaccine antibody response of the standard and high dose vaccine for three stains of influenza. (B) Histogram of maxRBA for both vaccine doses with dashed lines representing the median of the scores.

Vaccine responsiveness was expressed two ways: first, as the log fold-change vaccine antibody response, which was significantly greater for HD compared to SD recipients for all vaccine antigens, A/H1N1 (log fold-change difference = 0.37, *p* < 0.001), A/H3N2 (0.47, p < 0.001) and B (0.36, p < 0.001) (Fig. 2A); second, as a responsiveness index across all antigens (maxRBA), which was also significantly higher for HD recipients (standardized maxRBA difference = 0.61, p < 0.001) (Fig. 2B).

**Fig. 3.**
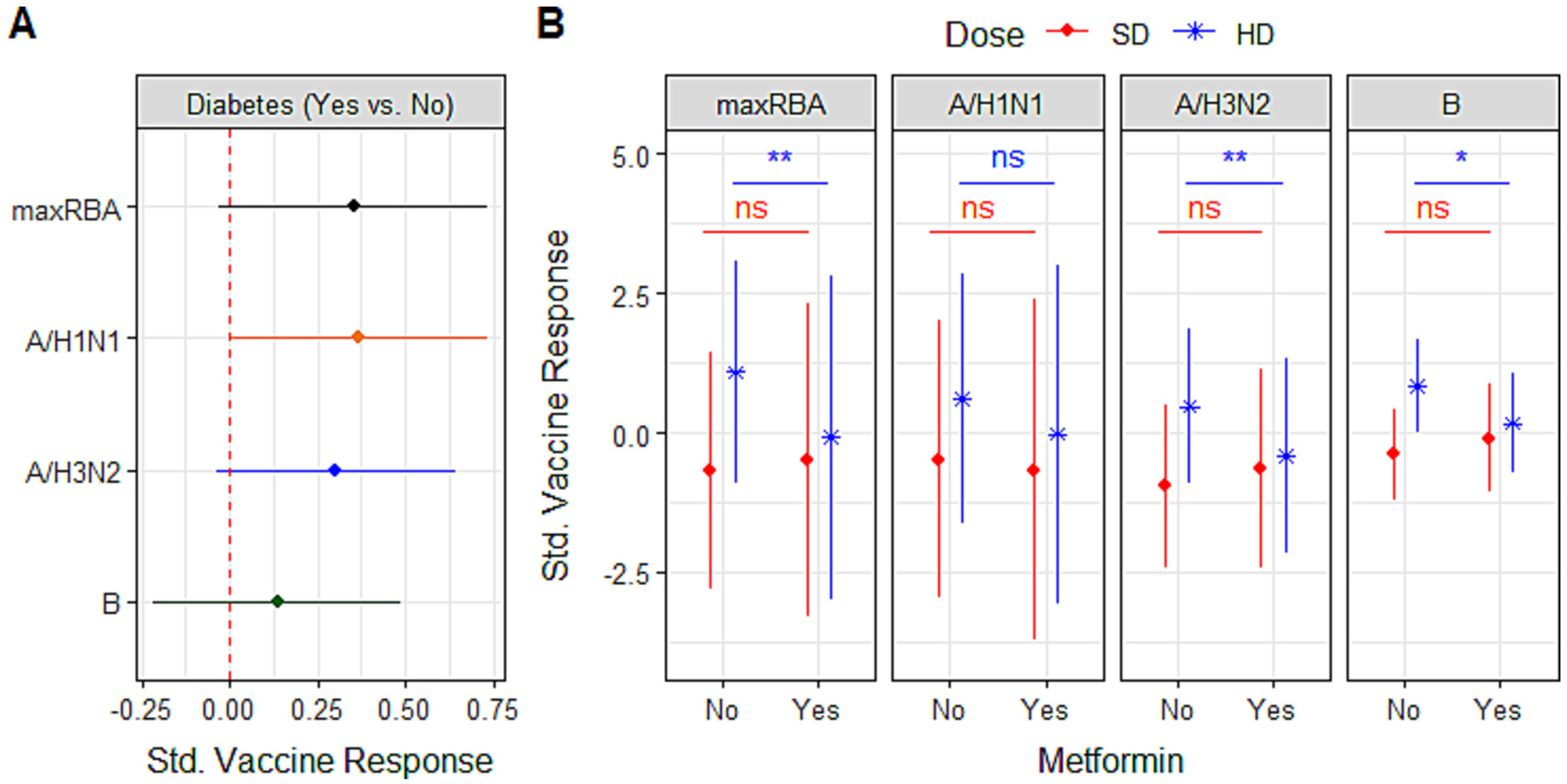
Dose-dependent associations between vaccine responsiveness and metformin usage within diabetic participants. (A) Standardized maxRBA or antigen-specific associations with diabetes status in fully adjusted models, reported as the regression coefficient and 95 % confidence interval (CI). (B) Within diabetic participants, the estimated marginal means and 95 % CI of each vaccine response measure for metformin users (*n* = 64) and non-users (*n* = 30) in presented while considering the interaction with vaccine dose received (standard or high dose). Statistical significance reported between dose groups as: *, p < 0.05; **, p < 0.01; ns, not significant.

To investigate the association between medication usage and vaccine responsiveness, we used multivariable linear regression, adjusting for participant age, sex, frailty, dose, and CMV serostatus (Table 2). Vaccine antibody responses were significantly higher in HD vaccine recipients, CMV seronegative participants, and frail as compared to robust participants, which we have previously shown [15]. Regarding total medication count, there were no significant differences in the standardized vaccine response across usage categories (5–9 vs. 0–4: coefficient [95 % CI] = −0.06 [−0.28, 0.15]; 10+ vs. 0–4 = 0.05 [−0.33, 0.43]). Similarly, for models including metformin or statin use, no associations with vaccine response were observed (metformin use, yes vs. no = −0.05 [−0.35, 0.25]; statin use, yes vs. no = −0.01 [−0.30, 0.11]). Given that primary indications and other chronic conditions that often coincide with medication usage may contribute to vaccine responsiveness, we also tested associations to metformin while adjusting for diabetes, and statins while adjusting for hyperlipidemia or hypertension. Although in neither case were associations to metformin or statins statistically significant (Supplementary Table 2), estimates for diabetes were notable, suggesting improved responses for diabetics, mainly for influenza A antigens (maxRBA, 0.35 [−0.03, 0.74], *p* = 0.069; A/H1N1 = 0.37 [−0.01, 0.73], *p* = 0.052; A/H3N2 = 0.30 [−0.04, 0.64], *p* = 0.081) (Fig. 3A).

Given previous evidence indicating that correlates of vaccine antibody responsiveness and effectiveness may differ according to both sex and vaccine dose, we also investigated those variables as potential modifiers using interaction analysis [15,21,22]. However, associations between medication usage (i.e. total count, metformin or statin use) and vaccine responsiveness (i.e. maxRBA or antigen-specific responses) within females or males (Supplementary Fig. 1), or vaccine dose recipient groups (Supplementary Fig. 2) failed to reach statistical significance. Since the majority of participants taking either metformin or statins were also diagnosed with diabetes or hyperlipidemia, respectively, we also performed stratified analyses for those chronic condition groups. Although vaccine responsiveness was not significantly associated with metformin usage in diabetics (*n* = 30 of 94), or statin usage in participants reporting hyperlipidemia (n = 30 of 251) (Supplementary Table 3), this appeared to be dependent on vaccine dose for diabetic metformin users. Specifically, the strong boosting effect of the high dose vaccine was mostly absent for metformin users vs. non-users for all response measures except A/H1N1 (high-dose recipient users vs. non-users: maxRBA, −0.08 vs 1.09, *p* = 0.002; A/H3N2, −0.42 vs. 0.48, *p* = 0.007; B, 0.17 vs. 0.83, *p* = 0.030) (Fig. 3B).

## 4. Discussion

Previous studies investigating the impact of medications on influenza vaccine responses have mostly focused on immunosuppressant use, most notably steroids and non-steroidal anti-inflammatory drugs, while more recent studies have investigated broad-spectrum antibiotics. In both cases, associations were not clear [10,23,24]. The goal of our study was to determine if medication count and class, specifically metformin and statins, were associated with vaccine antibody responses. However, our data showed that medication use was not broadly associated with vaccine responses. This is consistent with a recent study reporting influenza vaccine effectiveness to be similar between patients taking statins compared to those who were not and others showing that statins were not associated with the vaccine response [13,25–27]. However, our findings are in disagreement with two studies suggesting immunogenicity decreases with statin use [10,28]. Previous studies on metformin have indicated both positive and negative associations [10,29].

Although we did not observe medication usage to be broadly associated with vaccine responsiveness, we did observe correlations within relevant strata. When considering all participants in our cohort, we found that diabetes was associated with an increased, albeit non-significant increase in vaccine responsiveness while adjusting for metformin usage. This is supported by previous work reporting diabetes to be associated with improved influenza vaccine immunogenicity [30] and effectiveness of the pneumococcal vaccine [31]. However, there remains much debate regarding the relationship between diabetes and influenza vaccine responses [32], given that many studies fail to see any differences between diabetics and non-diabetics [32–34]. Since all but one metformin user was non-diabetic, we investigated a potential modifying role of vaccine dose in the response of diabetic participants. For non-metformin users, we observed a notable increase in post-vaccination antibody titres in high-dose as compared to standard-dose recipients, which is consistent with effects we observed for non-diabetic participants. However, the response for high-dose vaccine metformin users was significantly muted, and was only slightly higher than standard-dose vaccine metformin users. This observation was not expected, especially given the recent evidence suggesting metformin may improve vaccine immunogenicity [11,29]. To the best of our knowledge, no studies have compared the effect of vaccine dose according to metformin usage, although recent findings from experimental work support our findings. Metformin has been shown to suppress IFN-alpha expression in both whole blood and monocytes stimulated with poly I:C (i.e. TLR3 activation), or whole or split influenza virus preparations [35], and type I IFN signaling has been shown to be a central component to the boosting effect of high-dose quadrivalent vaccination [36]. Together, these data warrant further investigation into the potential effect of metformin across different vaccine formulations, particularly in diabetics. While it appears that high-dose vaccine recipients taking metformin exhibit immunogenicity that is no lower than standard-dose recipients, it is nonetheless important to confirm whether other enhanced vaccines such as adjuvanted formulations should instead be recommended for diabatic metformin users.

There is limited data on the impact of total medication use (i.e. polypharmacy) and vaccine responsiveness in older adults. One recent paper found that hyperpolypharmacy (10 or more medications/day) was associated with a higher antibody decline six months post-vaccination in residents living in long-term care facilities compared to those with no polypharmacy (0–4 medications/day) [37]. In our study, we found that participants taking 10+ medications compared to those taking only 0–4 medications exhibited higher antibody responses, although this was limited to HD vaccine recipients and failed to reach statistical significance. This possible synergism of medication count and vaccine dose echoes our previous work on this cohort, where frailty and biological age were significantly associated with increased responses to the HD vaccine, more so than the SD [15,16]. Given that medication use is much more common in frail older adults, it is possible that our observations are simply due to residual confounding that we were unable to account for in our regression model when adjusting for participant frailty class and chronic condition count. It is also plausible that the prior effects of frailty and biological age that we observed are mediated in part by polypharmacy, which we unfortunately cannot address in the current study.

Our study had several strengths and some limitations. We were able to investigate the effect of medication usage on antibody responses in a relatively large sample of older adults over the course of multiple influenza seasons, where year-to-year differences in vaccine formulation and baseline titers were accounted for using an effective baseline adjustment approach (i.e. maxRBA) and through random intercepts in our modelling. Additionally, we explored both SD and HD influenza vaccine recipients and controlled for important confounders, such as age, sex, frailty and CMV serostatus. However, our data was limited in scope as only prescription medications were considered without any further information regarding dose or non-prescription medications. Furthermore, this was a secondary analysis of study data not originally designed to investigate medication usage. As with other observational studies, many of our participants were on multiple medications, and it is possible that their metformin and/or statin usage could have been confounded by other concomitant medications, which we could not control for. Similarly, we were unable to tease apart the potential confounding effects of chronic conditions, namely hyperlipidemia, diabetes, or overall multimorbidity. Hence, the lack of significant estimates for our associations between medication usage and vaccine responsiveness may be due to our inability to account for variation due to chronic condition status.

## 5. Conclusions

In summary, we show that medication use may influence vaccine efficacy, but this effect is dependent on dose, antigen measured and disease status. Future studies involving larger cohorts and a randomized study design will likely be needed to help inform physicians of potentially harmful or beneficial off target effects of medication usage in older adults.

## Supplementary Material

1

## Figures and Tables

**Table 1:** Summary of participant characteristics.

	Total	2015/16	2016/17	2017/18	2018/19
	(*N* = 542)	(*N* = 175)	(*N* = 174)	(*N* = 155)	(*N* = 38)
**Age**					
Mean (SD)	76.7 (7.41)	75.6 (7.34)	76.7 (7.43)	77.1 (7.06)	80.6 (7.85)
Median [Min, Max]	76.0 [65.0, 97.0]	74.0 [65.0, 95.0]	76.0 [65.0, 95.0]	76.0 [65.0, 96.0]	81.5 [66.0, 97.0]
**Sex**					
Female	361 (66.6 %)	119 (68.0 %)	116 (66.7 %)	102 (65.8 %)	24 (63.2 %)
Male	181 (33.4 %)	56 (32.0 %)	58 (33.3 %)	53 (34.2 %)	14 (36.8 %)
**Vaccine**					
Standard dose	262 (48.3 %)	90 (51.4 %)	89 (51.1 %)	83 (53.5 %)	0 (0 %)
High dose	280 (51.7 %)	85 (48.6 %)	85 (48.9 %)	72 (46.5 %)	38 (100 %)
**Site**					
HSNRI	280 (51.7 %)	100 (57.1 %)	99 (56.9 %)	81 (52.3 %)	0 (0 %)
UCHC	262 (48.3 %)	75 (42.9 %)	75 (43.1 %)	74 (47.7 %)	38 (100 %)
**Body-mass index**					
Normal/Underweight	165 (30.4 %)	45 (25.7 %)	50 (28.7 %)	48 (31.0 %)	22 (57.9 %)
Overweight	224 (41.3 %)	83 (47.4 %)	79 (45.4 %)	51 (32.9 %)	11 (28.9 %)
Obese	153 (28.2 %)	47 (26.9 %)	45 (25.9 %)	56 (36.1 %)	5 (13.2 %)
**CMV serostatus**					
Negative	255 (47.0 %)	91 (52.0 %)	77 (44.3 %)	71 (45.8 %)	16 (42.1 %)
Positive	287 (53.0 %)	84 (48.0 %)	97 (55.7 %)	84 (54.2 %)	22 (57.9 %)
**Fried frailty phenotype**					
Robust	173 (31.9 %)	69 (39.4 %)	66 (37.9 %)	31 (20.0 %)	7 (18.4 %)
Pre-frail	323 (59.6 %)	93 (53.1 %)	96 (55.2 %)	109 (70.3 %)	25 (65.8 %)
Frail	46 (8.5 %)	13 (7.4 %)	12 (6.9 %)	15 (9.7 %)	6 (15.8 %)
**Chronic conditions (count)**					
0	78 (14.4 %)	19 (10.9 %)	21 (12.1 %)	29 (18.7 %)	9 (23.7 %)
1	136 (25.1 %)	46 (26.3 %)	39 (22.4 %)	42 (27.1 %)	9 (23.7 %)
2	114 (21.0 %)	32 (18.3 %)	38 (21.8 %)	34 (21.9 %)	10 (26.3 %)
3	99 (18.3 %)	33 (18.9 %)	36 (20.7 %)	24 (15.5 %)	6 (15.8 %)
4+	115 (21.2 %)	45 (25.7 %)	40 (23.0 %)	26 (16.8 %)	4 (10.5 %)
**Diabetes**					
Yes	94 (17.3 %)	27 (15.4 %)	34 (19.5 %)	30 (19.4 %)	3 (7.9 %)
No	448 (82.7 %)	148 (84.6 %)	140 (80.5 %)	125 (80.6 %)	35 (92.1 %)
**Hyperlipidemia**					
Yes	251 (46.3 %)	89 (50.9 %)	85 (48.9 %)	64 (41.3 %)	13 (34.2 %)
No	291 (53.7 %)	86 (49.1 %)	89 (51.1 %)	91 (58.7 %)	25 (65.8 %)
**Hypertension**					
Yes	303 (55.9 %)	104 (59.4 %)	98 (56.3 %)	79 (51.0 %)	22 (57.9 %)
No	239 (44.1 %)	71 (40.6 %)	76 (43.7 %)	76 (49.0 %)	16 (42.1 %)

*Abbreviations*: HSNRI, Health Sciences North Research Institute; UCHC, UConn Health.

**Table 2:** Associations between vaccine responsiveness expressed as the standardized maxRBA and medication usage in fully adjusted linear regression models.

		Medication count	Metformin use	Statin use
Medication count	0–4	REF		
	5–9	−0.06 (−0.28, 0.15)		
	10+	0.05 (−0.33, 0.43)		
	No		REF	REF
Status	Yes		−0.05 (−0.35, 0.25)	−0.1 (−0.3, 0.11)
Age	per decade	−0.11 (−0.25, 0.02)	−0.11 (−0.25, 0.02)	−0.12 (−0.25, 0.01)
	Female	REF	REF	REF
Sex	Male	−0.14 (−0.34, 0.07)	−0.14 (−0.34, 0.07)	−0.12 (−0.32, 0.08)
	Standard dose	REF	REF	REF
Vaccine	High dose	0.62 (0.46, 0.79)***	0.62 (0.46, 0.79)***	0.62 (0.46, 0.79)***
	Negative	REF	REF	REF
CMV serostatus	Positive	−0.25 (−0.44, −0.06)**	−0.25 (−0.44, −0.05)*	−0.24 (−0.43, −0.05)*
	Robust	REF	REF	REF
	Pre-frail	0.1 (−0.1, 0.3)	0.1 (−0.09, 0.3)	0.1 (−0.1, 0.29)
Fried frailty phenotype	Frail	0.34 (−0.01, 0.69)	0.33 (−0.01, 0.68)	0.34 (−0.01, 0.69)
	0	REF	REF	REF
	1	−0.03 (−0.33, 0.26)	−0.04 (−0.33, 0.25)	−0.01 (−0.31, 0.28)
	2	−0.13 (−0.44, 0.18)	−0.14 (−0.44, 0.16)	−0.1 (−0.42, 0.21)
Chronic conditions (count)	3	−0.08 (−0.41, 0.25)	−0.1 (−0.41, 0.22)	−0.04 (−0.38, 0.3)
	4+	0.25 (−0.11, 0.6)	0.25 (−0.07, 0.57)	0.3 (−0.03, 0.63)

Estimates represent the regression coefficient and 95 % confidence interval relative to the reference group (REF)or denoted units.

Statistical significance is indicated as

*, *p* <0.05,

**, *p* <0.01,

***, *p* <0.001.

## Data Availability

Data will be made available on request.

## Appendix A. Supplementary data

Supplementary data to this article can be found online at https://doi.org/10.1016/j.vaccine.2025.127913.

## References

[1] Naquin A, O’Halloran A, Ujamaa D, Sundaresan D, Masalovich S, Cummings CN, Laboratory-confirmed influenza-associated hospitalizations among children and adults - influenza hospitalization surveillance network, United States, 2010–2023. MMWR Surveill Summ 2024;73:1–18. 10.15585/mmwr.ss7706a1. PMC11537671 39471107

[2] Coll PP, Costello VW, Kuchel GA, Bartley J, McElhaney JE. The prevention of infections in older adults: vaccination. J Am Geriatr Soc 2020;68:207–14. 10.1111/jgs.16205. 31613000

[3] Kwong JC, Schwartz KL, Campitelli MA, Chung H, Crowcroft NS, Karnauchow T, Acute myocardial infarction after laboratory-confirmed influenza infection. N Engl J Med 2018;378:345–53. 10.1056/NEJMoa1702090. 29365305

[4] Covinsky KE, Palmer RM, Fortinsky RH, Counsell SR, Stewart AL, Kresevic D, Loss of independence in activities of daily living in older adults hospitalized with medical illnesses: increased vulnerability with age. J Am Geriatr Soc 2003;51:451–8. 10.1046/j.1532-5415.2003.51152.x. 12657063

[5] Wilkinson K, Wei Y, Szwajcer A, Rabbani R, Zarychanski R, Abou-Setta AM, Efficacy and safety of high-dose influenza vaccine in elderly adults: a systematic review and meta-analysis. Vaccine 2017;35:2775–80. 10.1016/j.vaccine.2017.03.092. 28431815

[6] Ferdinands JM, Thompson MG, Blanton L, Spencer S, Grant L, Fry AM. Does influenza vaccination attenuate the severity of breakthrough infections? A narrative review and recommendations for further research. Vaccine 2021;39:3678–95. 10.1016/j.vaccine.2021.05.011. 34090700

[7] Zimmermann P, Curtis N. Factors that influence the immune response to vaccination. Clin Microbiol Rev 2019;32:e00084–18. 10.1128/CMR.00084-18. 30867162 PMC6431125

[8] Qato DM, Wilder J, Schumm LP, Gillet V, Alexander GC. Changes in prescription and over-the-counter medication and dietary supplement use among older adults in the United States, 2005 vs 2011. JAMA Intern Med 2016;176:473–82. 10.1001/jamainternmed.2015.8581. 26998708 PMC5024734

[9] Yen F-S, Wei JC-C, Shih Y-H, Hsu CY, Hsu C-C, Hwu C-M. Metformin use before influenza vaccination may lower the risks of influenza and related complications. Vaccines (Basel) 2022;10:1752. 10.3390/vaccines10101752. 36298617 PMC9609450

[10] Agarwal D, Schmader KE, Kossenkov AV, Doyle S, Kurupati R, Ertl HCJ. Immune response to influenza vaccination in the elderly is altered by chronic medication use. Immun Ageing 2018;15:19. 10.1186/s12979-018-0124-9. 30186359 PMC6119322

[11] Martin DE, Cadar AN, Panier H, Torrance BL, Kuchel GA, Bartley JM. The effect of metformin on influenza vaccine responses in nondiabetic older adults: a pilot trial. Immun Ageing 2023;20:18. 10.1186/s12979-023-00343-x. 37131271 PMC10152024

[12] McLean HQ, Chow BDW, VanWormer JJ, King JP, Belongia EA. Effect of statin use on influenza vaccine effectiveness. J Infect Dis 2016;214:1150–8. 10.1093/infdis/jiw335. 27471318 PMC5034952

[13] Chung H, Campitelli MA, Buchan SA, Campigotto A, Chen B, Crowcroft NS, Evaluating the impact of statin use on influenza vaccine effectiveness and influenza infection in older adults. Clin Infect Dis 2023;77:303–11. 10.1093/cid/ciad148. 36942534 PMC10371308

[14] Wu F, Wang C, Li S, Ye Y, Cui M, Liu Y, Association between statins administration and influenza susceptibility: a systematic review and Meta-analysis of longitudinal studies. Viruses 2024;16:278. 10.3390/v16020278. 38400053 PMC10893112

[15] Loeb N, Andrew MK, Loeb M, Kuchel GA, Haynes L, McElhaney JE, Frailty is associated with increased hemagglutination-inhibition Titers in a 4-year randomized trial comparing standard- and high-dose influenza vaccination. Open Forum Infect Dis 2020:7. 10.1093/ofid/ofaa148. PMC7255647 32500087

[16] Verschoor CP, Belsky DW, Ma J, Cohen AA, Griffith LE, Raina P. Comparing biological age estimates using domain-specific measures from the Canadian longitudinal study on aging. J Gerontol A Biol Sci Med Sci 2020. 10.1093/gerona/glaa151. PMC7812432 32598446

[17] Fried LP, Tangen CM, Walston J, Newman AB, Hirsch C, Gottdiener J, Frailty in older adults: evidence for a phenotype. J Gerontol A Biol Sci Med Sci 2001;56:M146–56. 11253156 10.1093/gerona/56.3.m146

[18] Proulx J Drug use among seniors in Canada, 2016. Value Health 2018:21. 10.1016/J.JVAL.2018.04.1003.

[19] Research C for DE and. General Drug Categories FDA; 2018.

[20] Avey S, Mohanty S, Chawla DG, Meng H, Bandaranayake T, Ueda I, Seasonal variability and shared molecular signatures of inactivated influenza vaccination in young and older adults. J Immunol 2020;204:1661–73. 10.4049/jimmunol.1900922. 32060136 PMC7755271

[21] Picard E, Armstrong S, Andrew MK, Haynes L, Loeb M, Pawelec G, Markers of systemic inflammation are positively associated with influenza vaccine antibody responses with a possible role for ILT2(+)CD57(+) NK-cells. Immun Ageing 2022;19:26. 10.1186/s12979-022-00284-x. 35619117 PMC9134679

[22] Chambers C, Skowronski DM, Rose C, Serres GD, Winter A-L, Dickinson JA, Should sex be considered an effect modifier in the evaluation of influenza vaccine effectiveness? Open Forum Infect Dis 2018;5:ofy211. 10.1093/ofid/ofy211. 30263903 PMC6143149

[23] Saleh E, Moody MA, Walter EB. Effect of antipyretic analgesics on immune responses to vaccination. Hum Vaccin Immunother 2016;12:2391–402. 10.1080/21645515.2016.1183077. 27246296 PMC5027726

[24] Hagan T, Cortese M, Rouphael N, Boudreau C, Linde C, Maddur MS, Antibiotics-driven gut microbiome perturbation alters immunity to vaccines in humans. Cell 2019;178:1313–1328.e13. 10.1016/j.cell.2019.08.010. 31491384 PMC6750738

[25] Cutrell JB, Drechsler H, Bedimo R, Alvarez CA, Mansi IA. Statin use and medically attended acute respiratory illness among influenza vaccine recipients. Vaccine 2019;37:6707–13. 10.1016/j.vaccine.2019.09.024. 31543418

[26] MacIntyre CR, Chughtai AA, Das A, Rahman B, Moa AM, Gan CH, Effect of statin use on the risk of influenza and influenza vaccine effectiveness. Int J Cardiol 2021;332:205–8. 10.1016/j.ijcard.2021.03.055. 33775795

[27] Havers FP, Chung JR, Belongia EA, McLean HQ, Gaglani M, Murthy K, Influenza vaccine effectiveness and statin use among adults in the United States, 2011–2017. Clin Infect Dis 2019;68:1616–22. 10.1093/cid/ciy780. 30371753 PMC6495015

[28] Black S, Nicolay U, Del Giudice G, Rappuoli R. Influence of statins on influenza vaccine response in elderly individuals. J Infect Dis 2016;213:1224–8. 10.1093/infdis/jiv456. 26516142

[29] Diaz A, Romero M, Vazquez T, Lechner S, Blomberg BB, Frasca D. Metformin improves in vivo and in vitro B cell function in individuals with obesity and Type-2 diabetes. Vaccine 2017;35:2694–700. 10.1016/j.vaccine.2017.03.078. 28392139 PMC5560851

[30] Feery BJ, Hartman LJ, Hampson AW, Proietto J. Influenza immunization in adults with diabetes mellitus. Diabetes Care 1983;6:475–8. 10.2337/diacare.6.5.475. 6400708

[31] Huijts SM, van Werkhoven CH, Bolkenbaas M, Grobbee DE, Bonten MJM. Post-hoc analysis of a randomized controlled trial: diabetes mellitus modifies the efficacy of the 13-valent pneumococcal conjugate vaccine in elderly. Vaccine 2017;35:4444–9. 10.1016/j.vaccine.2017.01.071. 28410813

[32] Verstraeten T, Fletcher MA, Suaya JA, Jackson S, Hall-Murray CK, Scott DA, Diabetes mellitus as a vaccine-effect modifier: a review. Expert Rev Vaccines 2020;19:445–53. 10.1080/14760584.2020.1760098. 32516066

[33] Haq K, Fulop T, Tedder G, Gentleman B, Garneau H, Meneilly GS, Cytomegalovirus seropositivity predicts a decline in the T cell but not the antibody response to influenza in vaccinated older adults independent of type 2 diabetes status. J Gerontol A Biol Sci Med Sci 2017;72:1163–70. 10.1093/gerona/glw216. 27789617 PMC5861868

[34] McElhaney JE, Garneau H, Camous X, Dupuis G, Pawelec G, Baehl S, Predictors of the antibody response to influenza vaccination in older adults with type 2 diabetes. BMJ Open Diabetes Res Care 2015;3:e000140. 10.1136/bmjdrc-2015-000140. PMC4611872 26504526

[35] Saenwongsa W, Nithichanon A, Chittaganpitch M, Buayai K, Kewcharoenwong C, Thumrongwilainet B, Metformin-induced suppression of IFN-α via mTORC1 signalling following seasonal vaccination is associated with impaired antibody responses in type 2 diabetes. Sci Rep 2020;10:3229. 10.1038/s41598-020-60213-0. 32094377 PMC7039947

[36] Bonduelle O, Delory T, Franco-Moscardini I, Ghidi M, Bennacer S, Wokam M, Boosting effect of high-dose influenza vaccination on innate immunity among elderly. JCI Insight 2025;10:e184128. 10.1172/jci.insight.184128. 40036077 PMC12016920

[37] Trevisan C, Haxhiaj L, Malara A, Abbatecola A, Fedele G, Palmieri A, Polypharmacy and antibody response to SARS-CoV-2 vaccination in residents of long-term care facilities: the GeroCovid vax study. Drugs Aging 2023;40:1133–41. 10.1007/s40266-023-01075-9. 37938521

