# Supplementary material for: Medication count, including statin or metformin use, is not associated with influenza vaccine responses in older adults
Source: Vaccine. Author manuscript; Available in PMC 2026 Jun 22. (PMC13285864; doi:10.1016/j.vaccine.2025.127913)

**SUPPLEMENTARY DATA**

**Supplementary Table 1:** Summary statistics describing frequency of medication class usage per participant (participant usage) and total usage in the cohort.

|  | **Participant usage** | | **Total** | |
| --- | --- | --- | --- | --- |
|  | **Count** | **Frequency (%)^X^** | **Count** | **Frequency (%)^Y^** |
| Antihypertensives | 277 | 51.1 | 349 | 13.4 |
| Antithrombic Agents | 300 | 55.4 | 331 | 12.7 |
| Lipid Lowering Agents | 274 | 50.6 | 295 | 11.3 |
| Gastrointestinal Agents | 156 | 28.8 | 183 | 7.0 |
| Antiarrhythmics | 150 | 27.7 | 178 | 6.8 |
| Antidiabetic Agents | 85 | 15.7 | 135 | 5.2 |
| Thyroid Drugs | 123 | 22.7 | 131 | 5.0 |
| Respiratory Agents | 65 | 12.0 | 127 | 4.9 |
| Diuretics | 115 | 21.2 | 126 | 4.8 |
| Analgesics | 87 | 16.1 | 114 | 4.4 |
| Genitourinary Tract Agents | 89 | 16.4 | 100 | 3.8 |
| Antidepressants | 85 | 15.7 | 91 | 3.5 |
| Anti-Infectives | 55 | 10.1 | 72 | 2.8 |
| Ophthalmic Agents | 39 | 7.2 | 53 | 2.0 |
| Anxiolytics, Sedatives & Hypnotics | 47 | 8.7 | 49 | 1.9 |
| Anticonvulsants | 34 | 6.3 | 42 | 1.6 |
| Hormone Modulators | 41 | 7.6 | 41 | 1.6 |
| Corticosteroids | 33 | 6.1 | 34 | 1.3 |
| Osteoporotic Agents | 31 | 5.7 | 31 | 1.2 |
| Alzheimer’s or Parkinson's Disease Agents | 23 | 4.2 | 26 | 1.0 |
| Antihistamines | 23 | 4.2 | 23 | 0.9 |
| Sex Hormones | 18 | 3.3 | 21 | 0.8 |
| Antianginal Agents | 14 | 2.6 | 18 | 0.7 |
| Antipsychotics | 10 | 1.8 | 10 | 0.4 |
| Anti-Vertigo Agents | 6 | 1.1 | 6 | 0.2 |
| Ion Exchange Resins | 3 | 0.6 | 3 | 0.1 |
| Antimigraine Agents | 3 | 0.6 | 3 | 0.1 |
| Erythropoiesis-Stimulating Agents | 2 | 0.4 | 2 | 0.1 |
| Muscle Relaxants | 2 | 0.4 | 2 | 0.1 |
| Antineoplastics | 2 | 0.4 | 2 | 0.1 |
| Antiemetics | 1 | 0.2 | 1 | 0.0 |
| Retinoids | 1 | 0.2 | 1 | 0.0 |
| Antirheumatics | 1 | 0.2 | 1 | 0.0 |
| Stimulants | 1 | 0.2 | 1 | 0.0 |

^X^, represents the frequency of patients taking at least one of the denoted medication classes

^Y^, represents the frequency of that medications within the denoted class relative to all medications recorded

**Supplementary Table 2:** Associations between metformin or statin usage and vaccine responsiveness while adjusting for diabetes, hyperlipidemia, or hypertension status.

| **INCL. DIABETES** | **scl.maxRBA** | | **sH1N1.FC** | | **H3N2.FC** | | **B.FC** | |
| --- | --- | --- | --- | --- | --- | --- | --- | --- |
| *Predictors* | *Estimates* | *CI* | *Estimates* | *CI* | *Estimates* | *CI* | *Estimates* | *CI* |
| Age dec | -0.1 | -0.24 – 0.04 | 0.08 | -0.07 – 0.22 | -0.03 | -0.15 – 0.09 | -0.06 | -0.19 – 0.07 |
| Sex [Male] | -0.11 | -0.30 – 0.09 | -0.1 | -0.30 – 0.10 | -0.04 | -0.22 – 0.13 | -0.16 | -0.34 – 0.02 |
| Dose [High dose] | **0.63** | **0.47 – 0.80** | **0.66** | **0.51 – 0.82** | **0.37** | **0.21 – 0.52** | **0.5** | **0.34 – 0.66** |
| Fried cat [Pre-frail] | 0.11 | -0.09 – 0.31 | 0.02 | -0.16 – 0.21 | 0.1 | -0.08 – 0.28 | 0.15 | -0.03 – 0.33 |
| Fried cat [Frail] | **0.37** | **0.03 – 0.72** | 0.18 | -0.14 – 0.50 | 0.08 | -0.23 – 0.39 | **0.48** | **0.16 – 0.80** |
| CMV [Positive] | **-0.24** | **-0.43 – -0.05** | **-0.19** | **-0.38 – -0.00** | -0.15 | -0.32 – 0.01 | **-0.19** | **-0.37 – -0.02** |
| Diabetes [Yes] | 0.35 | -0.03 – 0.74 | 0.37 | -0.00 – 0.73 | 0.3 | -0.04 – 0.64 | 0.14 | -0.22 – 0.49 |
| Metformin cat [Yes] | -0.27 | -0.71 – 0.17 | -0.27 | -0.70 – 0.15 | -0.13 | -0.52 – 0.27 | -0.1 | -0.50 – 0.30 |
| H1N1 V1 [log] |  |  | **-0.3** | **-0.40 – -0.20** |  |  |  |  |
| H3N2 V1 [log] |  |  |  |  | **-0.22** | **-0.30 – -0.14** |  |  |
| B V1 [log] |  |  |  |  |  |  | **-0.33** | **-0.43 – -0.23** |
| **INCL.  HYPERLIPIDEMIA** | **scl.maxRBA** | | **sH1N1.FC** | | **H3N2.FC** | | **B.FC** | |
| *Predictors* | *Estimates* | *CI* | *Estimates* | *CI* | *Estimates* | *CI* | *Estimates* | *CI* |
| Age dec | -0.11 | -0.25 – 0.03 | 0.07 | -0.07 – 0.21 | -0.03 | -0.15 – 0.09 | -0.06 | -0.19 – 0.07 |
| Sex [Male] | -0.07 | -0.28 – 0.13 | -0.08 | -0.29 – 0.12 | -0.03 | -0.21 – 0.15 | -0.15 | -0.33 – 0.03 |
| Dose [High dose] | **0.63** | **0.46 – 0.79** | **0.65** | **0.50 – 0.81** | **0.36** | **0.21 – 0.52** | **0.5** | **0.34 – 0.65** |
| Fried cat [Pre-frail] | 0.13 | -0.07 – 0.32 | 0.04 | -0.15 – 0.22 | 0.12 | -0.06 – 0.29 | 0.16 | -0.02 – 0.34 |
| Fried cat [Frail] | **0.41** | **0.06 – 0.75** | 0.19 | -0.14 – 0.51 | 0.09 | -0.22 – 0.40 | **0.51** | **0.18 – 0.83** |
| CMV [Positive] | **-0.22** | **-0.40 – -0.03** | -0.17 | -0.36 – 0.01 | -0.14 | -0.30 – 0.03 | **-0.18** | **-0.35 – -0.01** |
| Hyperlipidemia [Yes] | 0.16 | -0.13 – 0.44 | 0.21 | -0.05 – 0.47 | 0.17 | -0.08 – 0.43 | -0.01 | -0.27 – 0.25 |
| Statin cat [Yes] | -0.17 | -0.46 – 0.11 | -0.15 | -0.43 – 0.12 | -0.07 | -0.33 – 0.18 | -0.02 | -0.29 – 0.24 |
| H1N1 V1 [log] |  |  | **-0.31** | **-0.41 – -0.21** |  |  |  |  |
| H3N2 V1 [log] |  |  |  |  | **-0.23** | **-0.31 – -0.16** |  |  |
| B V1 [log] |  |  |  |  |  |  | **-0.33** | **-0.43 – -0.23** |
| **INCL.  HYPERTENSION** | **scl.maxRBA** | | **sH1N1.FC** | | **H3N2.FC** | | **B.FC** | |
| *Predictors* | *Estimates* | *CI* | *Estimates* | *CI* | *Estimates* | *CI* | *Estimates* | *CI* |
| Age dec | -0.11 | -0.25 – 0.03 | 0.06 | -0.08 – 0.21 | -0.04 | -0.16 – 0.08 | -0.07 | -0.20 – 0.06 |
| Sex [Male] | -0.08 | -0.28 – 0.12 | -0.09 | -0.29 – 0.11 | -0.03 | -0.21 – 0.15 | -0.16 | -0.34 – 0.03 |
| Dose [High dose] | **0.63** | **0.46 – 0.79** | **0.66** | **0.50 – 0.81** | **0.37** | **0.21 – 0.52** | **0.49** | **0.33 – 0.65** |
| Fried cat [Pre-frail] | 0.13 | -0.07 – 0.32 | 0.03 | -0.15 – 0.22 | 0.12 | -0.06 – 0.30 | 0.15 | -0.03 – 0.33 |
| Fried cat [Frail] | **0.42** | **0.07 – 0.77** | 0.2 | -0.12 – 0.53 | 0.11 | -0.20 – 0.43 | **0.48** | **0.16 – 0.80** |
| CMV [Positive] | **-0.21** | **-0.40 – -0.02** | -0.18 | -0.36 – 0.01 | -0.13 | -0.30 – 0.04 | **-0.19** | **-0.36 – -0.02** |
| Hypertension [Yes] | 0.04 | -0.14 – 0.22 | 0.09 | -0.09 – 0.27 | 0 | -0.16 – 0.17 | 0.11 | -0.05 – 0.28 |
| Statin cat [Yes] | -0.06 | -0.24 – 0.13 | 0 | -0.19 – 0.18 | 0.06 | -0.11 – 0.22 | -0.04 | -0.21 – 0.13 |
| H1N1 V1 [log] |  |  | **-0.31** | **-0.41 – -0.21** |  |  |  |  |
| H3N2 V1 [log] |  |  |  |  | **-0.23** | **-0.31 – -0.15** |  |  |
| B V1 [log] |  |  |  |  |  |  | **-0.33** | **-0.43 – -0.22** |

Note: bolded estimates are considered statistically significant at p<0.05. Reference categories include: sex (female), dose (standard dose), Fried frailty (robust), CMV serostatus (negative), medication and chronic condition (no). V1 indicates the baseline visit.

**Supplementary Table 3:** Associations between metformin or statin usage and vaccine responsiveness in participants reporting diabetes, hyperlipidemia, or hypertension.

| **DIABETES PARTICIPANTS** | **scl.maxRBA** | | **sH1N1.FC** | | **sH3N2.FC** | | **sB.FC** | |
| --- | --- | --- | --- | --- | --- | --- | --- | --- |
| *Predictors* | *Estimates* | *CI* | *Estimates* | *CI* | *Estimates* | *CI* | *Estimates* | *CI* |
| Age (decade) | 0.18 | -0.30 – 0.66 | 0.01 | -0.46 – 0.48 | 0.2 | -0.25 – 0.64 | 0.18 | -0.18 – 0.53 |
| Sex [Male] | -0.27 | -0.82 – 0.28 | -0.2 | -0.70 – 0.30 | -0.16 | -0.68 – 0.35 | -0.09 | -0.50 – 0.33 |
| Dose [High dose] | **0.83** | **0.40 – 1.25** | **0.8** | **0.39 – 1.20** | **0.59** | **0.21 – 0.96** | **0.58** | **0.18 – 0.98** |
| Fried [Pre-frail] | 0.15 | -0.46 – 0.76 | 0.34 | -0.22 – 0.90 | 0.29 | -0.26 – 0.83 | -0.14 | -0.65 – 0.37 |
| Fried [Frail] | -0.36 | -1.27 – 0.56 | 0.06 | -0.78 – 0.91 | -0.45 | -1.25 – 0.35 | -0.1 | -0.87 – 0.67 |
| CMV [Positive] | -0.08 | -0.65 – 0.49 | -0.09 | -0.62 – 0.43 | 0.11 | -0.41 – 0.63 | **-0.55** | **-0.99 – -0.11** |
| Metformin [Yes] | -0.48 | -1.02 – 0.07 | -0.41 | -0.90 – 0.08 | -0.26 | -0.75 – 0.23 | -0.2 | -0.63 – 0.22 |
| H1N1 V1 [log] |  |  | **-0.51** | **-0.81 – -0.21** |  |  |  |  |
| H3N2 V1 [log] |  |  |  |  | **-0.46** | **-0.68 – -0.23** |  |  |
| B V1 [log] |  |  |  |  |  |  | **-0.43** | **-0.74 – -0.13** |
|  |  |  |  |  |  |  |  |  |
| **HYPERLIPIDEMIA PARTICIPANTS** | **scl.maxRBA** | | **sH1N1.FC** | | **sH3N2.FC** | | **sB.FC** | |
| *Predictors* | *Estimates* | *CI* | *Estimates* | *CI* | *Estimates* | *CI* | *Estimates* | *CI* |
| Age (decade) | 0.02 | -0.19 – 0.24 | 0.14 | -0.07 – 0.36 | 0.07 | -0.13 – 0.27 | 0.01 | -0.16 – 0.19 |
| Sex [Male] | -0.17 | -0.45 – 0.11 | -0.14 | -0.42 – 0.15 | -0.12 | -0.38 – 0.14 | -0.14 | -0.37 – 0.08 |
| Dose [High dose] | **0.68** | **0.43 – 0.93** | **0.7** | **0.47 – 0.93** | **0.4** | **0.17 – 0.62** | **0.41** | **0.20 – 0.62** |
| Fried [Pre-frail] | 0.19 | -0.11 – 0.49 | 0.18 | -0.11 – 0.47 | 0.2 | -0.07 – 0.47 | 0.24 | -0.00 – 0.49 |
| Fried [Frail] | **0.51** | **0.04 – 0.98** | **0.46** | **0.01 – 0.91** | 0.23 | -0.19 – 0.65 | **0.61** | **0.22 – 1.01** |
| CMV [Positive] | -0.24 | -0.52 – 0.05 | -0.23 | -0.51 – 0.05 | -0.15 | -0.41 – 0.11 | -0.22 | -0.45 – 0.01 |
| Statin [Yes] | 0.1 | -0.29 – 0.49 | -0.08 | -0.46 – 0.30 | 0.08 | -0.27 – 0.43 | 0.2 | -0.12 – 0.52 |
| H1N1 V1 [log] |  |  | **-0.31** | **-0.47 – -0.16** |  |  |  |  |
| H3N2 V1 [log] |  |  |  |  | **-0.23** | **-0.34 – -0.11** |  |  |
| B V1 [log] |  |  |  |  |  |  | **-0.33** | **-0.46 – -0.19** |
|  |  |  |  |  |  |  |  |  |

**Supplementary Figure 1:** Influenza vaccine responses by medication usage, stratified by sex via interaction analyses. Responses in females or males are represented by the maxRBA or antigen-specific responses 4-weeks post-vaccination, and presented as the estimated marginal mean and 95% confidence interval (CI). P-values (denoted above the upper CI when p≤0.15) represent the difference to the 0-4 medication group or “no” medication usage.


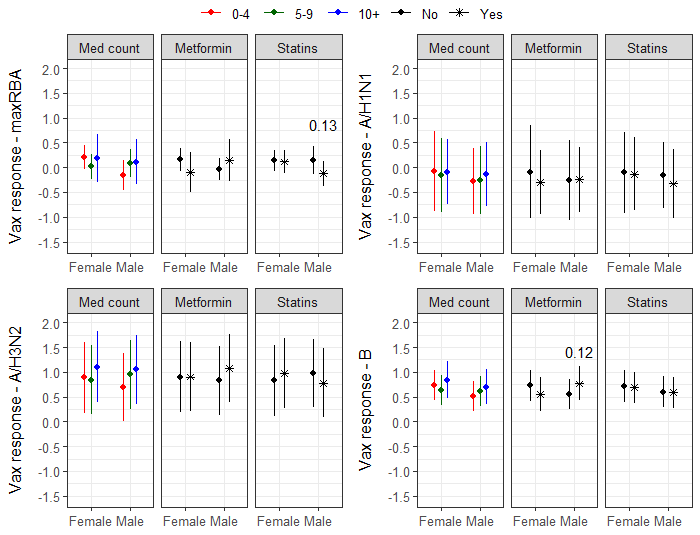


**Supplementary Figure 2:** Influenza vaccine responses by medication usage, stratified by dose via interaction analyses. Responses to the standard (SD) or high (HD) dose vaccine are represented by the maxRBA or antigen-specific responses 4-weeks post-vaccination, and presented as the estimated marginal mean and 95% confidence interval (CI). P-values (denoted above the upper CI when p≤0.15) represent the difference to the 0-4 medication group or “no” medication usage.


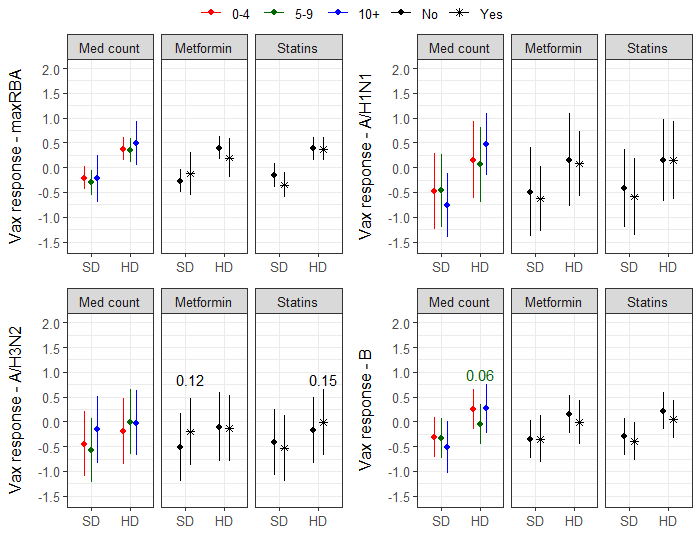

Supplement: 1 [file NIHMS2185386-supplement-1.docx]
